# A multiwavelength photoplethysmography dataset with blood pressure and heart rate reference measurements

**DOI:** 10.1016/j.dib.2025.112284

**Published:** 2025-11-14

**Authors:** Elisabetta Leogrande, Chiara Botrugno, Giulio Trono, Emanuele De Luca, Teresa Natale, Pasquale Tondo, Donato Lacedonia, Francesco Dell'Olio

**Affiliations:** a Department of Electrical and Information Engineering, Polytechnic University of Bari, Bari, 70126, Italy; b Department of Medical and Surgical Sciences, University of Foggia, 71122 Foggia, Italy; c Department of Specialistic Medicine, Pulmonary and Critical Care Unit, University-Hospital Polyclinic of Foggia, 71122 Foggia, Italy

**Keywords:** Photoplethysmography, Multiwavelength signals, Blood pressure estimation, Infrared and red light, Biomedical dataset

## Abstract

We present a dataset of photoplethysmographic (PPG) signals acquired from 127 seated subjects under controlled conditions. The signals were recorded using the MAX86150EVSYS, a commercial multiwavelength optical sensor capable of simultaneous acquisition at red and infrared wavelengths. Each signal was sampled at 100 Hz, following a standardized protocol that was formally reviewed and approved by the institutional ethics committee of Ospedali Riuniti di Foggia for biomedical data collection. In addition to the PPG waveforms, reference values of systolic and diastolic blood pressure, as well as heart rate, were obtained using a commercial sphygmomanometer. All measurements were performed while minimizing motion to ensure high-quality signals suitable for physiological modeling and algorithm development. This dataset is intended to support research in blood pressure estimation, PPG signal analysis, and the development of robust cardiovascular monitoring techniques for wearable health technologies. All data were collected with informed consent and in accordance with institutional ethical guidelines.

## Graphical abstract

**Figure:**
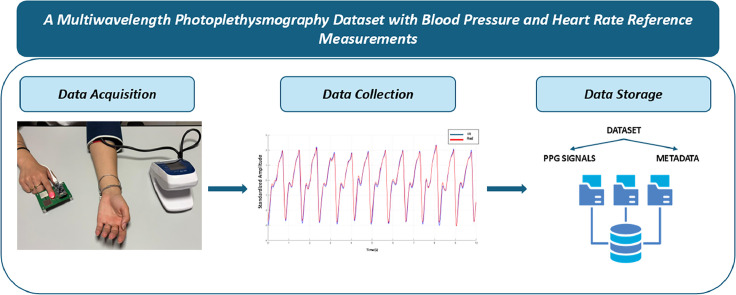

Specifications Table

**Table:** 

Subject	Health Sciences, Medical Sciences & Pharmacology
Specific subject area	Photoplethysmography-based cardiovascular monitoring
Type of data	MATLAB arrays, Text File
Data collection	Data were collected using the MAX86150EVSYS, a dual-wavelength optical sensor, during simultaneous acquisition of photoplethysmographic (PPG) signals and blood pressure measurements from 127 seated subjects. The PPG signals were recorded from the index finger at 100 Hz using DeviceStudio software. Reference systolic and diastolic blood pressure values, as well as heart rate, were obtained using a commercial sphygmomanometer. The dataset was curated by trimming each recording to 1000 samples (10 s). MATLAB was used for signal processing.
Data source location	Azienda Ospedaliero-Universitaria “Ospedali Riuniti”, Viale Luigi Pinto 1, 71122 Foggia, Italy
Data accessibility	Repository name: A Multiwavelength Photoplethysmography Dataset with Blood Pressure and Heart Rate Reference Measurements Data identification number: 10.17632/gs7jvwtcgt.1 Direct URL to data: https://data.mendeley.com/datasets/gs7jvwtcgt/1 Instructions for accessing these data: Publicly available; no restrictions
Related research article	[1] C. Botrugno and F. Dell’Olio, «PPG-Based Electronic Sensing System for Cuff-Less Calibration-Free Blood Pressure Estimation Using Machine Learning Algorithms», in 2024 IEEE Sensors Applications Symposium (SAS), Naples, Italy: IEEE, Jul. 2024, pp. 1–5. doi: 10.1109/SAS60918.2024.10636647.

## 1 Value of the Data

• The dataset includes synchronized red and infrared PPG signals along with reference measurements of systolic and diastolic blood pressure, enabling the development and testing of models for estimating blood pressure from optical signals. This supports the growing interest in cuffless, continuous blood pressure monitoring solutions.
• The controlled acquisition conditions and high-quality signals make this dataset suitable for validating signal preprocessing techniques, noise filtering methods, and wearable sensor technologies aimed at continuous monitoring of cardiovascular parameters.
• Researchers can extract both morphological and spectral features from clean PPG waveforms that are temporally aligned with reference blood pressure and heart rate values. These features are essential for physiological interpretation and diagnostic applications.
• The dataset structure, consistent acquisition protocol, and detailed metadata enable reproducibility in machine learning workflows. This makes it a valuable resource for developing, training, and benchmarking models for health parameter estimation and classification tasks.
• Beyond academic research, this dataset has strong potential for industrial and translational applications. It can support the validation of wearable devices and the development of telehealth platforms, as well as contribute to regolatory testing frameworks for medical devices. While each recording is limited to 10 s and thus not directly intended for continous monitoring applications, the dataset reamins highly valuable for developing and benchmarking algorithms for signal preprocessing, feature extraction, and blood pressure estimation, foundational steps that underpin continous cardiovascular monitoring solutions. Its standardized structure, high signal quality, and curated reference measurements make it a robust resource for accelerating the transition of photoplethysmography-based technologies from laboratory research to certified clinical products and commercial health solutions.

## 2 Background

The dataset was collected to investigate the relationship between PPG signal morphology and arterial blood pressure, enabling the development of machine learning models for non-invasive estimation of systolic and diastolic values. Data were obtained using a dual-wavelength optical sensor under a clinical protocol at Ospedali Riuniti di Foggia. This work builds upon validated non-invasive approaches: photoplethysmography has demonstrated accuracy in cuffless pressure estimation [2], while parameters derived from the digital volume pulse, such as pulse wave velocity, correlate strongly with arterial pressure [3]. Multimodal fusion of synchronized ECG and PPG recordings further enhances estimation precision by exploiting cross-signal features [4]. Embedded portable systems have been shown feasible for home monitoring [5], and advanced wavelet-based enhancement together with novel PPG feature selection significantly reduce estimation error [6]. The dataset encompasses diverse age ranges and clinical conditions, ensuring robustness and generalizability. Finally, widely used repositories - Photoplethysmography PPG Dataset [7], VitalDB [8], complementary multi-parameter waveform collections [9], and the Scientific Data PPG-BP Dataset [10] and the MIMIC database [11] - provide essential benchmarks for comparative validation.

## 3 Data Description

The dataset includes 127 PPG recordings acquired at 100 Hz from the index finger using the MAX86150EVSYS device. Each recording is 10 s long (1000 samples), with simultaneously measured systolic BP, diastolic BP, and heart rate. Data are stored in “.mat” format. The folder structure is described in the repository README file.

Table 1 summarizes the main acquisition parameters and protocol settings used in this study, including sensor characteristics, sampling conditions, and reference measurements.

**Table 1:** Summary of the technical and procedural parameters used during data acquisition.

Parameter	Value/Description
Number of subjects	127
Sampling frequency	100 Hz
Recording duration (per subject)	10 s (1000 samples)
PPG wavelenghts	Red and Infrared
Acquisition site	Index finger (reflective mode)
Device	MAX86150EVSYS
Acquisition software	DeviceStudio
Reference measurements	Systolic BP, Diastolic BP, Heart Rate
Measurement location	Ospedale Riuniti di Foggia
Protocol	Approved institutional protocol
Data format	Digital, exported via DeviceStudio
Recording conditions	Seated, resting, no motion

To assess the quality and variability of the recordings, basic descriptive statistics were computed for each PPG channel.

**Fig. 1:**
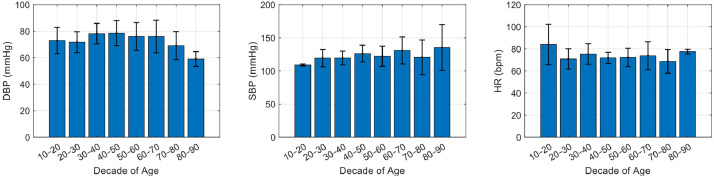
Mean values of DBP (on the left), SBP (in the middle) and HR (on the right) calculated over all 127 subjects and grouped by 10-year age intervals. Each bas represents the average ± standard deviation within a given age group. This visualization highlights age-related trends in cardiovascular parameters across the studied cohort.

**Fig. 2:**
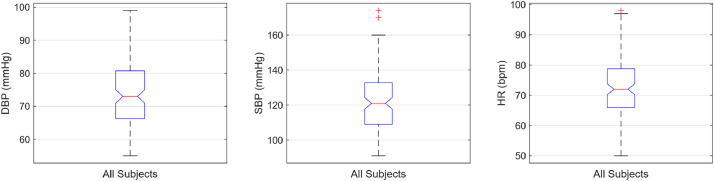
Boxplots showing the distributions of DBP (on the left), SBP (in the middle) and HR (on the right) across all 127 subjects. Boxes represent the interquartile range (IQR), horizontal lines indicate the median, and whiskers denote 1.5 x IQR. These distributions illustrate inter-subject variability and the overall physiological range of the dataset.

**Fig. 3:**
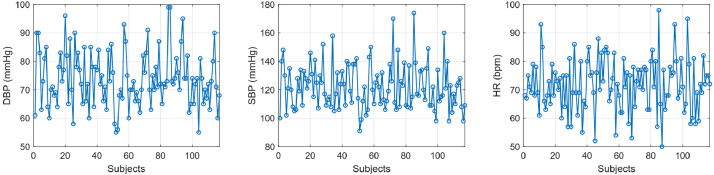
Individual trajectories of DBP (on the left), SBP (in the middle) and HR (on the right), for each subject. Each line corresponds to one subject’s measurement, showing inter-individual variability and highlighting the diversity of physiological values across the cohort.

The dataset includes 127 subjects aged between 18 and 86 years, divided into 64 females and 63 males, stratified by ten-year age groups as reported in Table 2. Table 3 shows the mean values of SBP, DBP, and HR for males and females, while Fig. 1 presents the mean physiological parameters (SBP, DBP, and HR) by age decade calculated over the entire cohort. The distributions of these physiological parameters for each individual subject can be seen in Fig. 1, Fig. 2, Fig. 3.

**Table 2:** Number of subjects in each ten­-year age interval, stratified by sex.

Sex	10–20	20–30	30–40	40–50	50–60	60–70	70–80	80–90
Females	2	32	4	1	15	7	2	1
Males	0	24	8	6	8	12	4	1

**Table 3:** Mean ± STD of age, SBP, DBP, and HR for female and male groups.

Sex	N subjects	Age mean ± STD	SBP mean ± STD	DBP mean ± STD	HR mean ± STD
Females	64	39.17 ± 18.22	117.39 ± 15.00	70.84 ± 8.69	74.03 ± 10.01
Males	63	42.89 ± 18.03	127.03 ± 15.24	76.90 ± 9.76	70.46 ± 8.96

The results of the time‐domain analysis highlighted statistically significant differences in PPG signal dynamics between males and females. In particular, the signal dynamics - defined as the difference between the maximum and minimum values of the standardized amplitude of the filtered PPG signal - showed mean values of 3.80 ± 0.71 for the infrared channel in females and 3.34 ± 1.04 in males, while for the red channel the mean values were 3.76 ± 0.84 in females and 3.27 ± 1.25 in males. Since the assumption of normality was not met for at least one subgroup, a nonparametric test was employed to compare differences between sexes. Using a nonparametric test to compare dynamics between males and females, a statistically significant difference emerged for both the infrared channel (*p* = 0.014) and the red channel (*p* = 0.045), confirming that the amplitude excursion of the PPG signal is on average more pronounced in females than in males. In Table 4, the mean values and their respective standard deviations are reported. These differences in signal dynamics may be partially attributed to known physiological variations, such as vascular compliance and skin thickness, which can influence light absorption and reflection in photoplethysmographic measurements. In particular, females often exhibit higher peripheral perfusion and thinner dermal layers, which may contribute to greater amplitude excursions in PPG signals [12].

**Table 4:** Mean ± STD of PPG signal dynamics (max–min of standardized amplitude) for infrared and red channels, by sex and for the total cohort.

	IR Dynamics Mean ± STD	R Dynamics Mean ± STD
Females	3.80 ± 0.71	3.76 ± 0.84
Males	3.34 ± 1.04	3.27 ± 1.25
Total	3.57 ± 0.91	3.52 ± 1.08

Regarding the PPG waveform shape, the skewness and kurtosis analysis on the entire cohort confirms that the distribution of signal values is on average symmetric and devoid of heavy tails. The mean skewness of the infrared channel is 0.063 ± 0.340, while that of the red channel is 0.057 ± 0.353. The kurtosis values are 1.965 ± 0.273 for infrared and 2.014 ± 0.308 for red. In Table 5, the mean values and their respective standard deviations are reported.

**Table 5:** Mean ± STD of skewness and kurtosis for infrared and red PPG signals, by sex and for the total cohort.

	Skewness IR (Mean ± STD)	Kurtosis IR (Mean ± STD)	Skewness RED (Mean ± STD)	Kurtosis RED (Mean ± STD)
Total	0.063 ± 0.340	1.965 ± 0.273	0.057 ± 0.353	2.014 ± 0.308

**Fig. 4:**
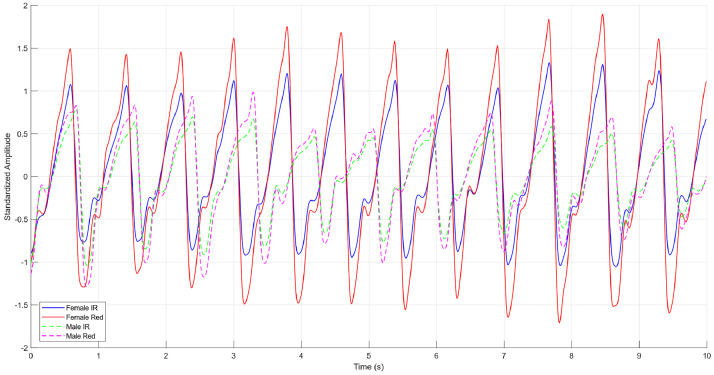
Representative photoplethysmographic waveforms acquired from one female subject (solid line) and one male subject (dotted line) in the IR and Red channels. The figure illustrates typical signal morphology and amplitude differences between the two channels, as well as sex-related differences in PPG signal dynamics.

The near-zero skewness values confirm that the PPG waveform, once filtered and standardized, tends to maintain a balanced morphology, without significant right or left tail asymmetry. Additionally, the sub-Gaussian kurtosis (values < 3) suggests a platykurtic distribution, indicative of a relatively flat peak and light tails, which is consistent with a physiological signal predominantly shaped by periodic cardiac cycles. These results show that, in most subjects, the PPG waveform exhibits a stable and symmetric pattern. In Fig. 4, an example of a PPG signal in the two channels (Infrared and Red) can be observed.

**Fig. 5:**
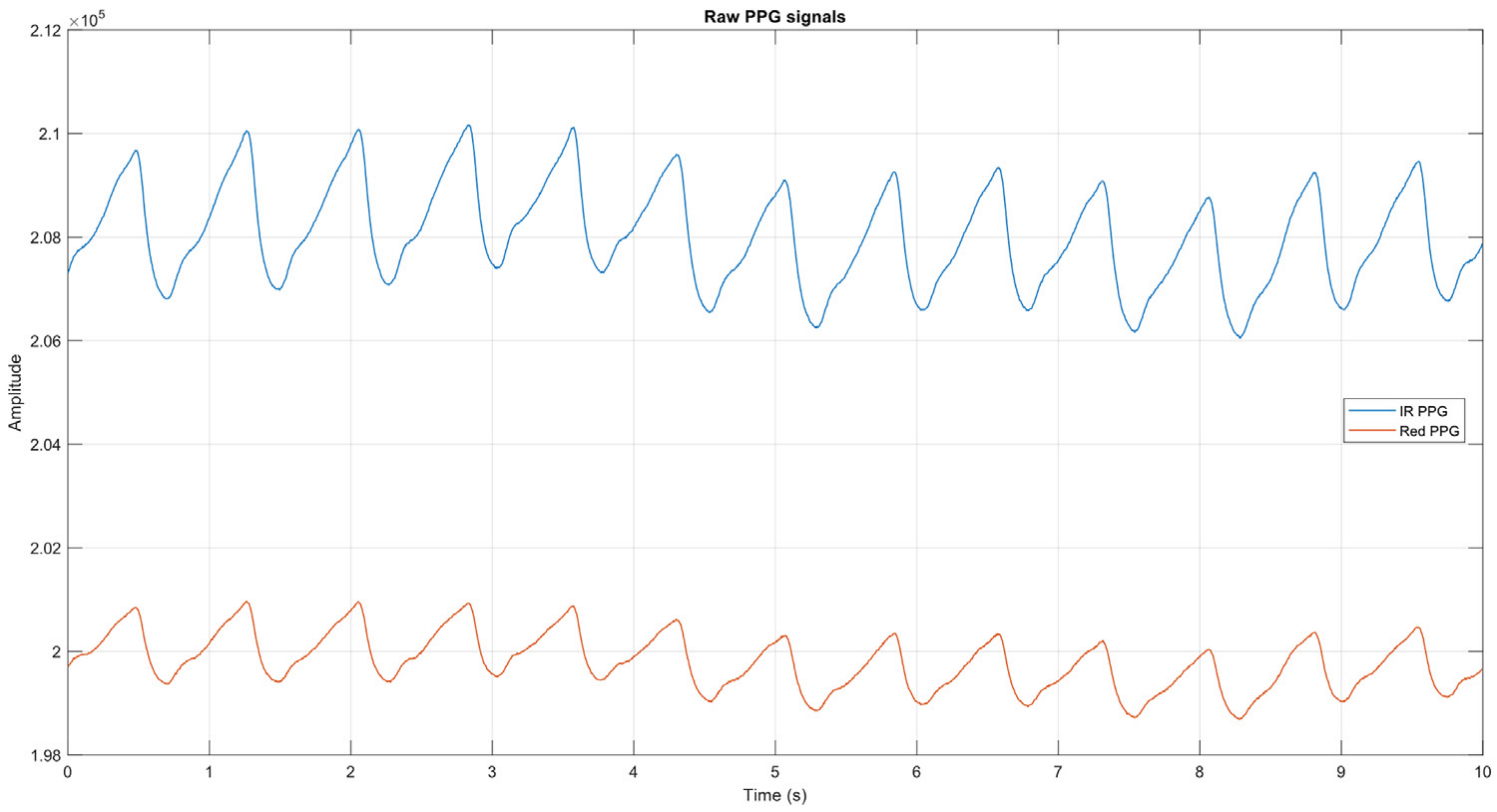
Representative example of raw infrared and red PPG signals prior to preprocessing.

To complement this view, Fig. 5 shows a representative example of raw infrared and red PPG signals prior to preprocessing. Although the raw signals appear relatively stable, they still require preprocessing to ensure suitability for analysis. Even without visually evident artifacts, subtle baseline fluctuations, transient outliers, or sampling inconsistencies can affect feature extraction and model performance if left unaddressed, underscoring the importance of preprocessing even when the raw waveform morphology seems visually acceptable.

**Fig. 6:**
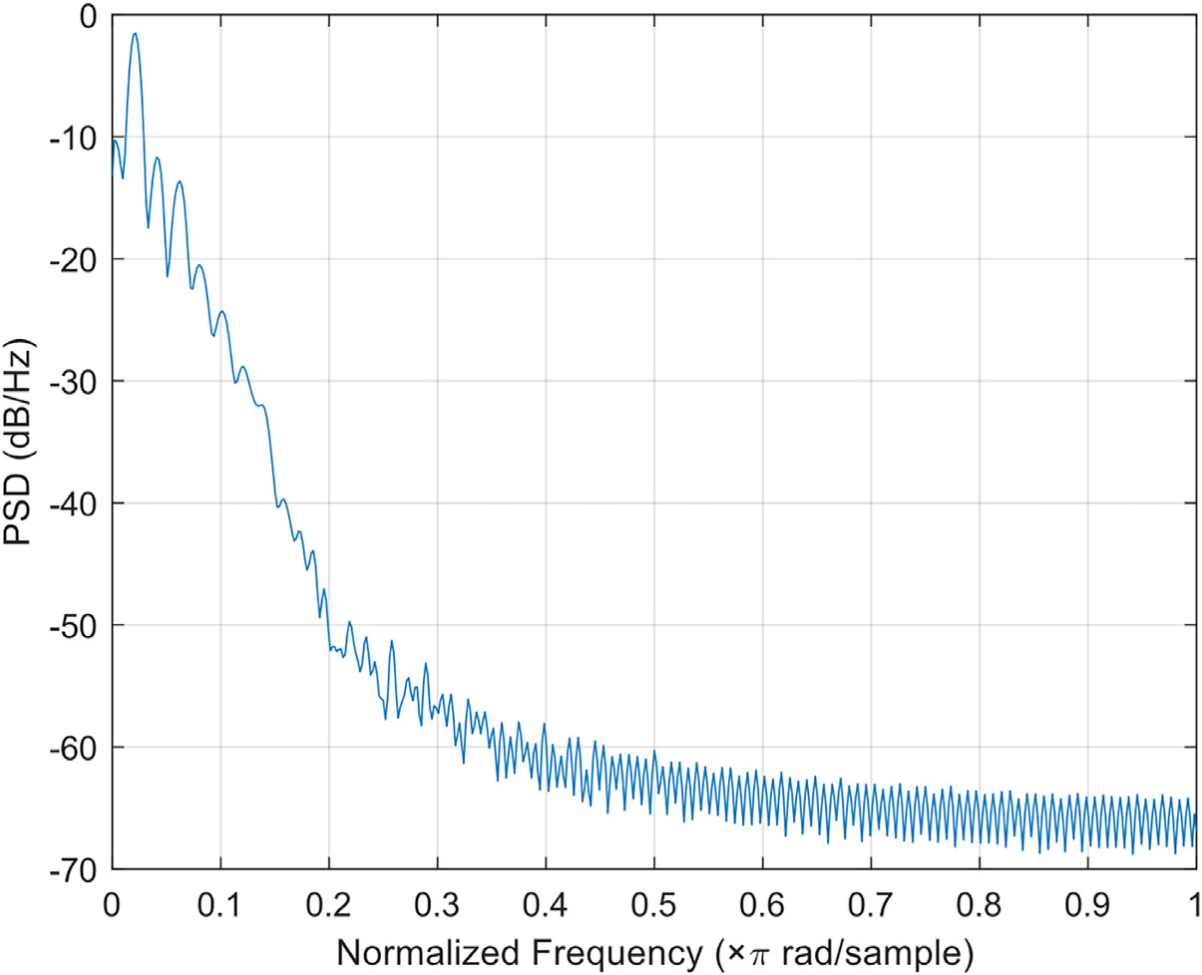
Power spectral density of a representative infrared PPG signal. A dominant peak is visible in the 1–2 Hz frequency range, corresponding to the cardiac component. Beyond this range, spectral power decreases rapidly, indicating the presence of weaker harmonics and residual noise.

To illustrate the spectral characteristics of a typical PPG recording in the infrared channel, we randomly selected one example signal, corresponding to index 10 in the array “input_data.mat”. To evaluate its frequency content, we applied two complementary techniques: power spectral density (PSD) estimation and time–frequency analysis via the spectrogram. As shown in Fig. 6, the PSD exhibits a clear peak between 1 and 2 Hz, which corresponds to the cardiac frequency band. Immediately following this peak, power drops rapidly to values around –40/–60 dB/Hz in the 2–10 Hz range, where only weaker harmonics and a reduced noise contribution remain.

**Fig. 7:**
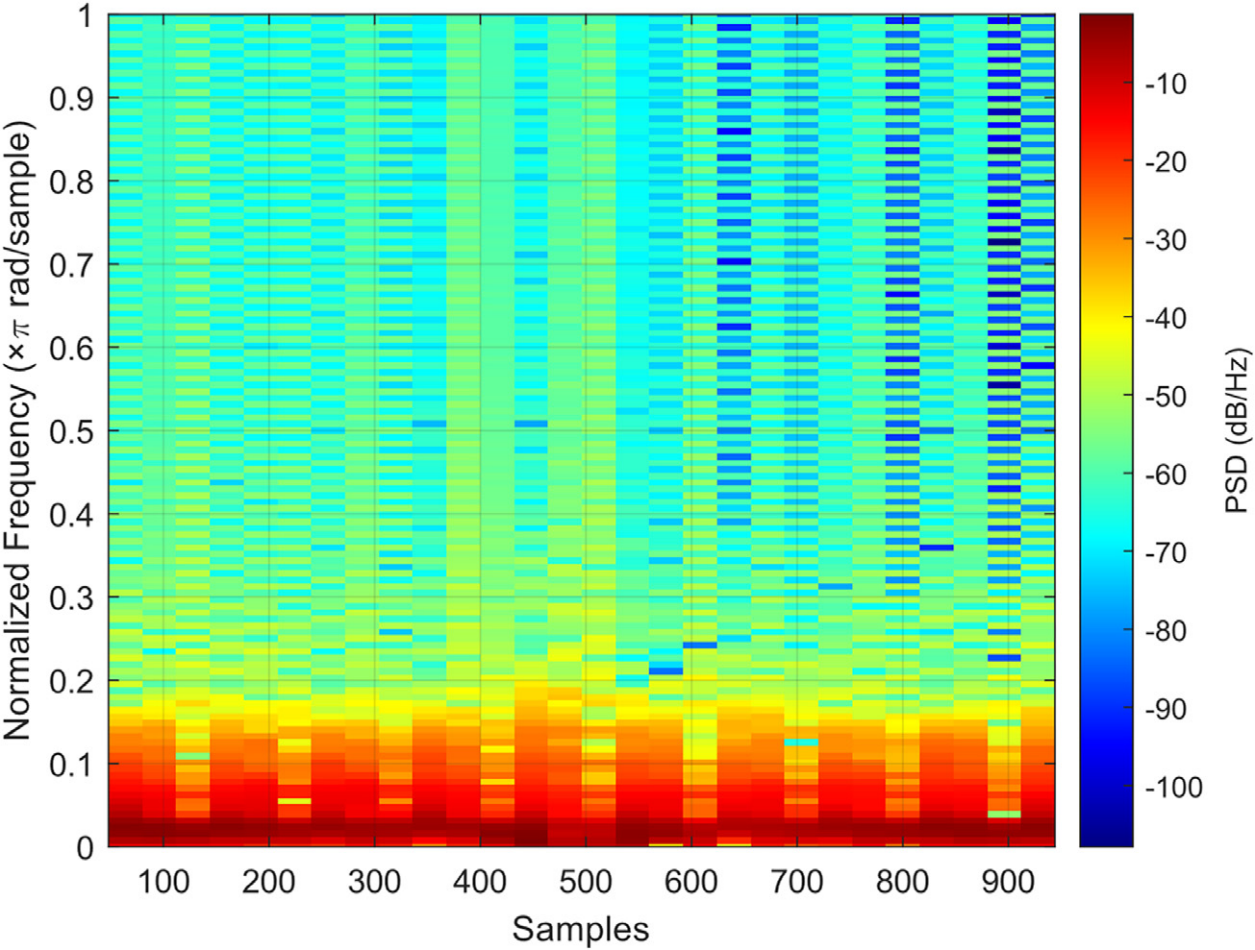
Spectrogram of the infrared PPG signal. The time-frequency representation shows that >50  % of the signal energy is concentrated in the cardiac band (1.0–2.25 Hz), while lower and higher frequencies contribute less. This confirms the stability and dominance of the cardiac component throughout the recording.

As illustrated in Fig. 7, >50 % of the useful energy (0.5–10 Hz) is concentrated in the cardiac band (1–2.25 Hz), while approximately 39 % remains between 0.5 and 1 Hz, and <1 % is distributed from 2.25 to 10 Hz. This distribution confirms that the cardiac component is dominant.

**Fig. 8:**
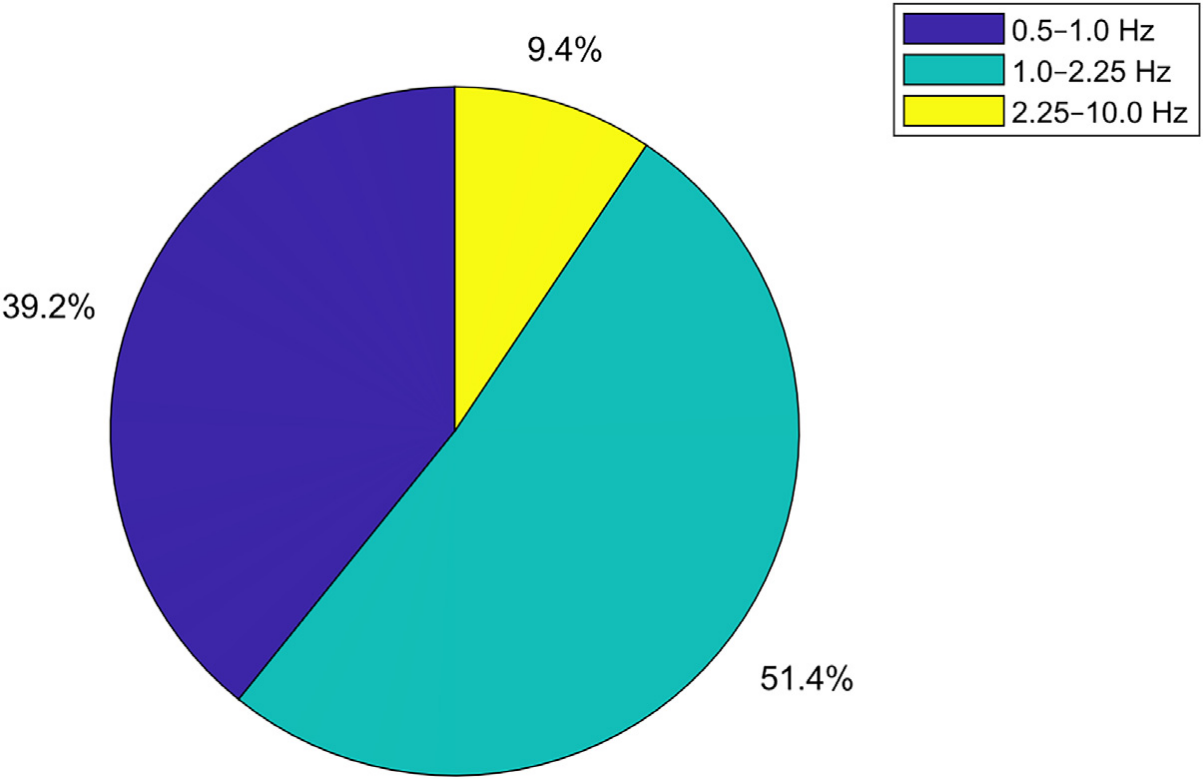
Percentage distribution of total PPG signal power across three physiological frequency bands: 0.5–1.0 Hz (low-frequency), 1.0–2.25 Hz (cardiac band), 2.25–10 Hz (high-frequency). The dominance of the cardiac band confirms the physiological relevance and quality of the recorded signals.

The spectrogram of the same signal, shown in Fig. 8, corroborates the PSD observations. In particular, the band of highest power remains consistently between 1 and 2 Hz throughout the entire recording, indicating a stable heart rate.

**Fig. 9:**
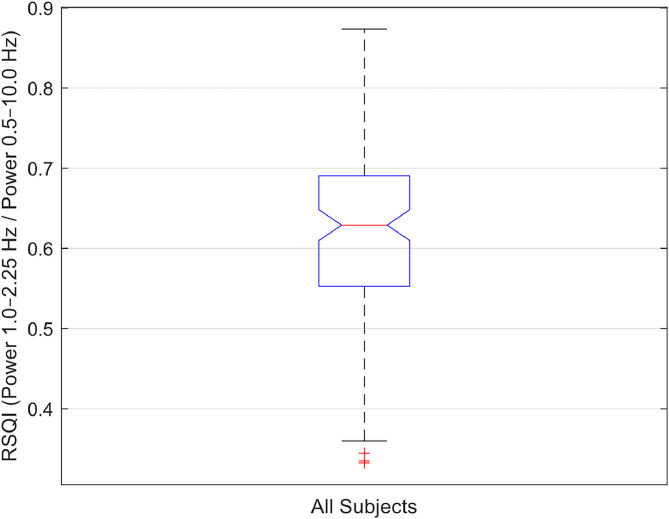
Boxplot of the Relative Signal Quality Index (RSQI) across all 127 subjects. The RSQI quantifies signal quality. The median value of approximately 0.63 indicates that most recordings have a strong cardiac component.

To assess the overall quality of the PPG signals in terms of spectral content, we computed, for each subject, the Relative Signal Quality Index (RSQI), defined as the ratio of the integrated power in the cardiac band (1.00–2.25 Hz) to the total power in the 0.50–10.00 Hz range. The RSQI serves as a practical index for evaluating signal usability, particularly in real-world or wearable device contexts where motion artifacts and noise can impair signal fidelity. A high RSQI ensures that the PPG signal retains a dominant and stable cardiac component, facilitating robust heart rate and variability analysis [13]. The RSQI boxplot across all 127 subjects, shown in Fig. 9, reveals that the median is approximately 0.63, indicating that, for half of the subjects, >63 % of the “useful” power (0.50–10.00 Hz) is concentrated in the 1.00–2.25 Hz band. The first quartile (Q1 ≈ 0.55) and third quartile (Q3 ≈ 0.69) delimit the central 50 % of values, confirming that most recordings are dominated by the cardiac component. In summary, the plot demonstrates that the cardiac component is predominant in most PPG recordings and allows immediate identification of subjects whose signal quality might be compromised. Overall, both time-domain and spectral analyses consistently highlight the reliability and physiological relevance of the recorded PPG signals.

Moreover, users of the dataset can apply thresholding based on the RSQI to automatically exclude low-quality recordings from their analyses. For example, selecting a threshold slightly below the median value (e.g., RSQI > 0.55) allows researchers to retain signals with a dominant cardiac component and discard those potentially affected by excessive noise or motion artifacts. This optional step can be particularly useful in preprocessing pipelines, ensuring that subsequent feature extraction and model training are performed on high-quality signals.

## 4 Experimental Design, Materials and Methods

**Fig. 10:**
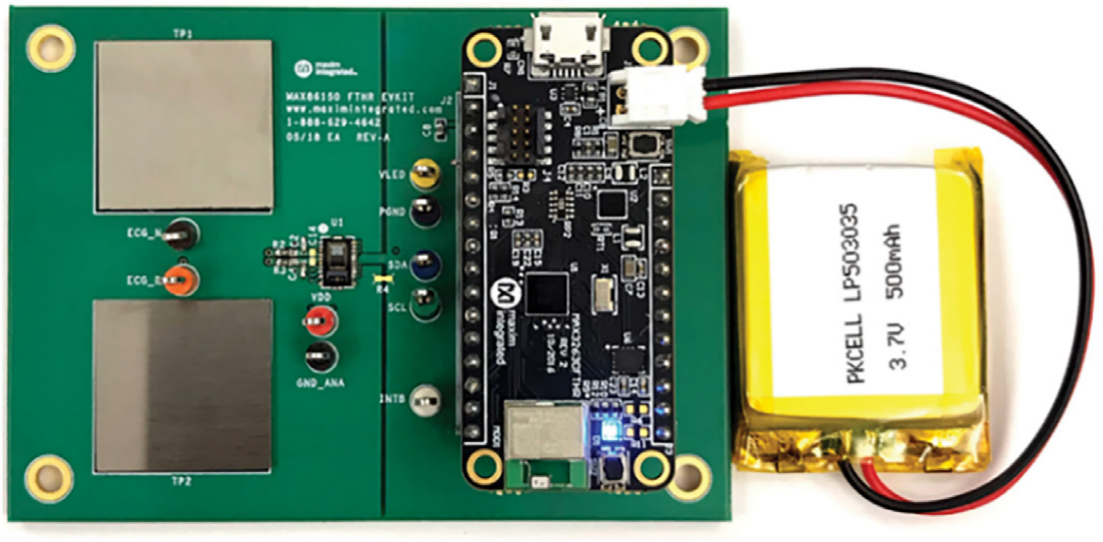
MAX86150EVSYS evaluation board used for dual-wavelength PPG acquisition. The board integrates red and infrared LEDs with a photodetector in reflective configuration, optimized for detecting pulsatile blood flow signals at the fingertip.

Participants placed their index finger on the MAX86150EVSYS sensor while seated and at rest. The fingertip was selected as the measurement site due to its anatomical and physiological suitability for photoplethysmographic (PPG) acquisition. This region is rich in capillary beds and exhibits strong pulsatile blood flow, making it ideal for capturing signals influenced by heartbeat dynamics, peripheral hemodynamics, and arteriole compliance. These factors contribute to characteristic waveform shapes that can reflect underlying cardiovascular conditions. Additionally, the fingertip offers a stable and accessible location with relatively uniform tissue thickness, which improves signal quality and reduces variability. The placement of the light-emitting diode (LED) and photodetector (PD) on the sensor is designed to optimize the detection of reflected light from arterial blood flow, enhancing both signal amplitude and robustness to motion. Among potential sites, the fingers are commonly preferred for PPG sensing due to the consistently high signal-to-noise ratio they provide compared to other body regions. Fig. 10 shows the MAX86150EVSYS evaluation board used for PPG acquisition, with integrated red and infrared optical components and a photodetector in reflective mode.

**Fig. 11:**
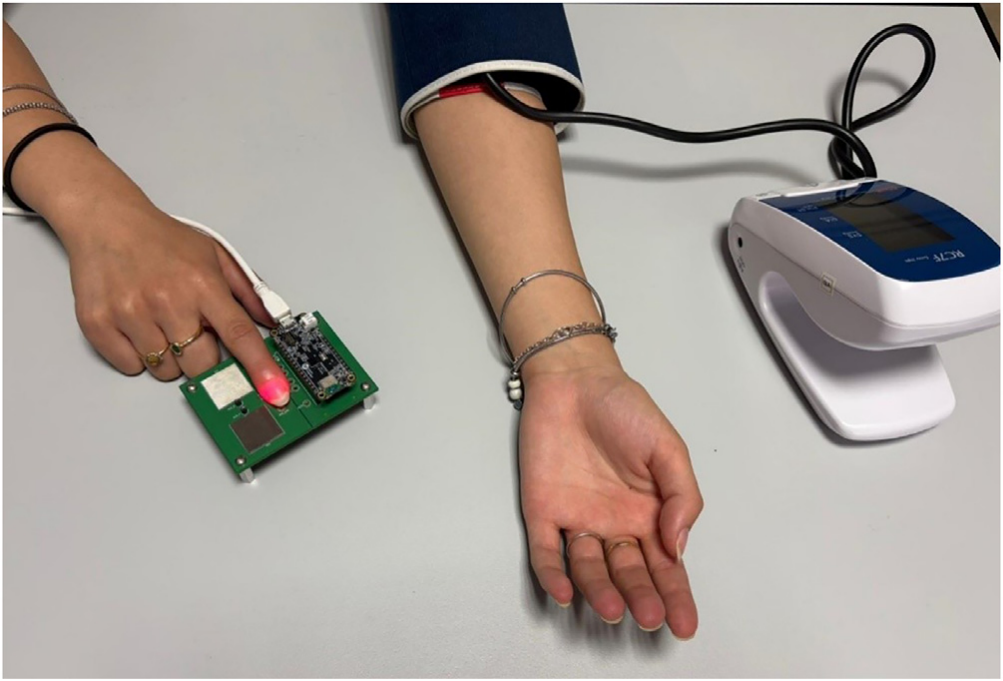
Experimental setup for synchronized acquisition of PPG signals and reference blood pressure values.

A commercial sphygmomanometer was placed on the contralateral arm, as shown in Fig. 11. The reference blood pressure measurements were obtained using a commercial digital oscillometric sphygmomanometer (Mediland RC7F). The device bears the CE mark and complies with the Medical Devices Directive 93/42/EEC, Annex V. It is classified as a Class II medical device, and the manufacturer operates a quality management system certified to EN ISO 13485:2016.

Simultaneous acquisition of PPG and reference measurements was performed using DeviceStudio.

Each session resulted in a 10-second PPG recording (1000 samples) and a reference triplet: systolic blood pressure, diastolic blood pressure, and heart rate. The choice to retain 1000 samples per recording was made to ensure consistency across the dataset and to capture sufficient physiological information within a short, standardized window. At the chosen sampling rate, data typically includes multiple cardiac cycles, allowing for reliable extraction of temporal and morphological features (e.g., pulse intervals, peak amplitudes, rise time). This duration balances the need for informative signal segments with practical considerations such as data size, ease of processing, and comparability between subjects. Additionally, a fixed-length window facilitates the use of machine learning algorithms that often require uniform input dimensions. The device operated in reflective mode using red and infrared LEDs. Signal data were saved in CSV format and processed using MATLAB for trimming and analysis. The study included normotensive and hypertensive subjects aged 18–86 years and followed a protocol approved by the local ethics committee.

## Limitations

While the dataset provides high-quality PPG signals collected under controlled conditions, some limitations must be acknowledged. First, data acquisition was performed exclusively on subjects with light skin tones, all belonging to the same ethnic background. As a result, the dataset may not fully capture the variability in PPG signal characteristics associated with different levels of melanin concentration or skin thickness, which are known to influence optical signal propagation.

## Ethics Statement

This study was conducted at Ospedali Riuniti di Foggia in accordance with the Declaration of Helsinki and was approved by the local institutional ethics committee (clinical‑ethics protocol No. 181/CE/2024, 17 December 2024). All participants provided written informed consent. Data were anonymised and managed in full compliance with GDPR (D.lgs. 101/2018).

## CRediT Author Statement

**E. Leogrande:** Data curation and Analysis, Writing – original draft. **C. Botrugno:** Data curation and Analysis. **G. Trono:** Data collection and Analysis. **E. De Luca:** Data curation and Analysis. **T. Natale:** Data curation and Analysis. **P. Tondo:** Investigation, Resources. **D. Lacedonia:** Investigation, Resources, Supervision. **F. Dell’Olio:** Supervision, Conceptualization, Writing – review.

## Acknowledgements

This research was supported by the authors’s affiliated institutions. The authors thank the clinical and technical staff of the Azienda Ospedaliero-Universitaria “Ospedali Riuniti” di Foggia for their support during data collection.

## Declaration of Competing Interest

The authors declare that they have no known competing financial interests or personal relationships that could have appeared to influence the work reported in this paper.

## Data Availability

Mendeley DataA Multiwavelength Photoplethysmography Dataset with Blood Pressure and Heart Rate Reference Measurements (Original data).

## Appendix Supplementary Materials

Image, application 1
